# Associations of genetic variants contributing to gut microbiota composition in diabetic nephropathy

**DOI:** 10.3389/fendo.2023.1264517

**Published:** 2023-12-20

**Authors:** Xiao Lu, Junjun Ma, Lili Guo, Wei Wu, Rongshan Li

**Affiliations:** ^1^ Department of Nephrology, Fifth Hospital of Shanxi Medical University (Shanxi Provincial People’s Hospital), Taiyuan, China; ^2^ Department of Thoracic Surgery, Fifth Hospital of Shanxi Medical University (Shanxi Provincial People’s Hospital), Taiyuan, China; ^3^ Shanxi Provincial Key Laboratory of Kidney Disease, Fifth Hospital of Shanxi Medical University (Shanxi Provincial People’s Hospital), Taiyuan, China; ^4^ Department of Clinical Laboratory, Fifth Hospital of Shanxi Medical University (Shanxi Provincial People’s Hospital), Taiyuan, China

**Keywords:** diabetic nephropathy, diabetes mellitus, susceptibility genes, gut microbiota, microbial quantitative trait locus

## Abstract

**Introduction:**

The gut microbiota is strongly associated with multiple kidney diseases, and since microbial composition is heritable, we hypothesized that genetic variations controlling gut microbiota composition were associated with diabetic nephropathy susceptibility or clinical subphenotypes.

**Methods:**

The genetic variations associated with gut microbiota were retrieved from the genome-wide association study database and analysed in our diabetic nephropathy susceptibility gene screening cohort. Candidate microorganisms with possible genetic associations were identified using the annotation of microbial quantitative trait loci. Finally, the candidate microorganisms were verified by 16S rDNA gene sequencing.

**Results:**

There were 13 genetic variation loci associated with susceptibility to diabetic nephropathy. The TCF7L2 risk genotype was associated with a long duration of diabetes and high diastolic blood pressure, the ZCWPW2 risk genotype was associated with increased glycosylated hemoglobin, and the ZNRF3 risk genotype was associated with an increased urinary microalbumin-to-creatinine ratio. Both the ZNRF3 and SPECC1L risk genotypes were associated with the abundance of Lactococcus. 16S rDNA sequencing confirmed that there was indeed a significant difference in the Lactococcus genus between DN and DM patients.

**Conclusions:**

In this study, we preliminarily confirmed that the gut microbiota of diabetic nephropathy patients is influenced by host genetics and provide a new basis for future accurate diagnosis and treatment.

## Introduction

Diabetic nephropathy (DN) is a serious complication of diabetes mellitus (DM) with high morbidity and mortality (1, 2). Approximately 30%-40% of patients with diabetes will develop diabetic nephropathy (3, 4), which has become the most common cause of end-stage renal disease (ESRD) in the world. The pathogenesis of DN is complex and is currently believed to be the result of the comprehensive action of multiple factors. Genetic factors have been found to play an important role (5). Recently, genome-wide association studies (GWAS) on diabetic nephropathy in different ethnic groups have reported some susceptibility sites (6–8).

There are approximately 500-1000 kinds of bacteria in the human gastrointestinal tract. The number of bacteria reaches 10^14^ colony-forming units and are called acquired “organs” (9). They assist the host in maintaining normal physiological functions. At present, a large number of studies have shown that the gut microbiota can participate in the occurrence and development of diseases by regulating host energy metabolism, the systemic inflammatory response, the secretion of enterogenic hormones and other mechanisms (10). The gut microbiota also plays an important role in a variety of kidney diseases, and the endotoxins, proteins and some metabolites produced by them have certain effects on the kidney through the gut-renal axis (11).

Genetic factors and microorganisms can influence the development of a wide range of complex diseases, but how precisely they interact in diabetic nephropathy is unclear. It has been shown that the composition of the gut microbiota is heritable and that host-microbe interactions play a role in the genetic architecture of several disease (12, 13). Therefore, in this study, we explored the role of gut microbiota in the etiology of diabetic nephropathy from the perspective of genetic susceptibility to diabetic nephropathy.

## Materials and methods

### SNP site selection

The NHGRI GWAS Catalogue (14) database was used to search for genetic variation loci associated with gut microbes until January 1, 2023, and to conduct quality control. The quality control criteria were as follows:

(1) SNP exclusion with loci deletion rate > 5%, (2) SNP loci with minimum allele frequency (MAF) ≤0.01 were excluded, and (3) SNP sites that deviate from Hardy-Weinberg equilibrium test (*P* < 0.0001) were excluded.

### Study subjects were included, and clinical indicators were collected.

We included 85 patients with diabetic nephropathy confirmed by renal biopsy and 107 patients with type 2 diabetes for more than 10 years without microvascular disease in Shanxi Provincial People’s Hospital from September 2019 to September 2022. In the DN group, the inclusion criteria were as follows: 18-65 y of age; diagnosed with type 2 diabetes; diabetic nephropathy diagnosed based on renal biopsy pathological examination; no evidence of primary renal disease; estimated glomerular filtration rate (eGFR)≥60 ml/min/1.73 m2; and signed informed consent.

In the T2DM group, the inclusion criteria were as follows: 18-65 y of age; the duration of type 2 diabetes was more than 10 years; no diabetic microvascular complications, including diabetic retinopathy and renal damage (eGFR≥60 ml/min/1.73 m2 and urine microalbumin-to-creatinine ratio, UACR<30 mg/g); and signed informed consent.

The exclusion criteria for both groups included the following: severe heart, lung, liver, kidney and other organ dysfunction; malignant tumor, autoimmune disease or psychiatric disorders; and pregnant or lactating women.

Demographic and clinical data, including age, sex, body mass index, duration of diabetes, blood pressure, urinary microalbumin-to-creatinine ratio, 24-hour urinary protein, fasting glucose, serum creatinine, glomerular filtration rate, glycated hemoglobin, and blood uric acid, were collected.

### DNA extraction and genotyping

Peripheral blood samples of patients were collected and placed in EDTA anticoagulant tubes. Genomic DNA was extracted by automatic nucleic acid extraction instrument, and genotyping was performed by the CAS-CN1 gene Chip. SNP quality control requirements were as follows: (1) the proportion of sample deletion sites >10% was excluded, (2) the deletion rate of the SNP site was > 10% was excluded, (3) SNP loci with minimum allele frequency (MAF) ≤0.01 were excluded, (4) Hardy-Weinberg balance test (HWE) *P* < 0.0001, excluding deviated indicators, and (5) sex examination.

### Fecal DNA extraction and 16S rDNA sequencing

We included 50 patients with diabetic kidney disease confirmed by renal biopsy (DN group) and 50 patients with type 2 diabetes for more than 10 years without microvascular disease (DM group) in Shanxi Provincial People’s Hospital from September 2019 to September 2022, with the same inclusion criteria as described above. The exclusion criteria for both groups included severe heart, lung, liver, kidney and other organ dysfunction; malignant tumor, autoimmune disease or gastrointestinal disease; the use of antibiotics, preparations of live bacteria, lactulose or immunosuppressants within nearly a month; and pregnant or lactating women.

Fresh fecal samples were obtained with a sterile fecal collector and the fecal samples were transferred to an ice box at -80°C within 2 hours after sampling. DNA was extracted from the samples using the QIAamp PowerFecal DNA Kit. Agarose gel electrophoresis was used to analyze DNA integrity. NanoDrop was used to measure the purity, and DNA concentration was accurately quantified with Qubit.

High-fidelity DNA polymerase was used to amplify the V3-V4 variable region of DNA by two-step PCR amplification and the addition of tag and joint sequences. An FC Magnetic Beads Kit (Enlighten) was used to purify and recover the product. Qubit4.0 was used to quantify the purified library. Qsep100 was used to check whether the length of the library was as expected, and each sample was diluted to 4 nM. The hybrid library was prepared and denatured by DNA, and at least a 5% Phix library was added to balance the library polymorphism. Sequencing was carried out on an Illumina MiSeq sequencer using the PE300 strategy.

### Ethics statement

All study procedures complied with the ethical guidelines of the Declaration of Helsinki. The studies involving human participants were reviewed and approved by the Biomedical Ethics Committee of Shanxi Provincial People’s Hospital (No. 2019-117). The patients/participants provided their written informed consent to participate in this study.

### 16S rDNA sequencing data processing and analysis

After disembarkation data filtering, the remaining high-quality clean data was obtained for later analysis. The reads were spliced into tags by the overlap between reads. The tags were clustered into OTUs, compared with the database, and species were annotated. Based on OTU and annotation results, sample species complexity analysis and intergroup species difference analysis was performed. Alpha diversity is used to analyze species diversity in a single sample, Beta diversity is used to compare the size of differences in species diversity between different samples. Wilcoxon rank sum tests (two tailed) were conducted to detect differences in relative abundances between the two groups.

### Statistical analysis

Plinks-1.07 was used for genetic association analysis (15). The ggpubr R package was used for mapping. The Wilcoxon rank sum test was used for comparisons between two groups, and the Kruskal-Wallis rank sum test was used for comparisons among multiple groups.

## Results

### General characteristics of the study participants

GWAS analysis was performed on 85 patients with DN and 107 patients with DM. The basic clinical characteristics of the case group and the control group were shown in **Table 1**. There were no significant differences in gender, body mass index, fasting blood glucose, glycosylated hemoglobin and blood pressure between the two groups. The age of DM group was significantly higher than that of DN group (*P*=0.007), and the duration of diabetes was longer than that of DN group (*P*=0.035). Serum creatinine level, urinary microalbumin-to-creatinine level, 24-hour urinary protein level and blood uric acid level in DN group were significantly increased than in DM group.

**Table 1:** General characteristics of participants in DN GWAS study.

	Total	DN	DM	P value
Sex, n (%)				>0.05
Male	140 (73)	62 (73)	78 (73)	
Female	52 (27)	23 (27)	29 (27)	
Age (years)	54.04 ± 8.8	51.19 ± 10.15	56.9 ± 6.08	0.007
BMI (kg/m2)	25.4 ± 3.04	25.87 ± 3.35	24.92 ± 2.66	0.153
Duration of DM (years)	11.87 ± 5.99	10.51 ± 6.23	13.23 ± 5.46	0.035
FBG (mmol/L)	8.16 ± 3.04	8.58 ± 3.75	7.75 ± 2.07	0.521
HbA1C (%)	8.53 ± 1.88	8.73 ± 2.04	8.33 ± 1.7	0.331
Scr (umol/L)	91.15 ± 60.62	114.76 ± 78.07	67.55 ± 14	< 0.001
GFR (ml/min*1.73m2)	94.96 ± 35.71	77.52 ± 35.43	112.4 ± 26.49	< 0.001
UACR (mg/g)	1497.26 ± 2304.45	2951.45 ± 2520.5	43.08 ± 248.59	< 0.001
24-hour urinary protein (g)	2.27 ± 3.31	4.34 ± 3.58	0.19 ± 0.8	< 0.001
Blood uric acid (umol/L)	352.04 ± 85.69	377.66 ± 93.51	326.42 ± 68.98	0.003
SBP (mmHg)	137.46 ± 19.46	142.42 ± 23.46	132.5 ± 12.83	0.063
DBP (mmHg)	82.17 ± 11.24	83.5 ± 12.1	80.83 ± 10.27	0.281

DN, diabetic nephropathy confirmed by renal biopsy; DM, type 2 diabetes mellitus for more than 10 years without kidney damage; BMI, body mass index; FBG, fasting blood glucose; HbA1c, glycosylated hemoglobin; Scr, serum creatinine; GFR, glomerular filtration rate; UACR, urine microalbumin-to-creatinine ratio; SBP, systolic blood pressure; DBP, diastolic blood pressure.

We included 50 patients with DN, 50 patients with DM to perform 16S rDNA sequence. The median age of the DN group was 51.52 years, and the mean duration of diabetes was 10.36 years. The median age of DM patients was 56.50 years and the mean duration of diabetes was 12.39 years (**Table 2**).

**Table 2:** General characteristics of participants in 16S rDNA sequence study.

	DN (n = 50)	DM (n = 50)	*P* value
Age (years)	51.52 ± 9.45	56.50 ± 6.28	0.018
Sex, n (%)			0.723
Female	14 (28)	16 (32)	
Male	36 (72)	34 (68)	
Duration of DM (years)	10.36 ± 6.49	12.39 ± 5.27	0.143
BMI (kg/m2)	25.94 ± 3.27	24.82 ± 2.79	0.73
Scr (umol/L)	96.34 ± 37.78	67.26 ± 14.02	< 0.001
FBG (mmol/L)	8.16 ± 2.42	7.72 ± 1.91	0.292
HbA1C (%)	9.1 ± 1.73	8.46 ± 1.83	0.934
UACR (mg/g)	2082.01 ± 2295.32	52.78 ± 279.34	< 0.001
GFR (ml/min/1.73m2)	86.33 ± 35.30	112.00 ± 27.07	0.002

DN, diabetic nephropathy confirmed by renal biopsy; DM, type 2 diabetes mellitus for more than 10 years without kidney damage; BMI, body mass index; Scr, serum creatinine; FBG, fasting blood glucose; HbA1c, glycosylated hemoglobin; UACR, urine microalbumin-to-creatinine ratio; GFR, glomerular filtration rate.

### Single nucleotide polymorphism results of DN GWAS

After quality control, a total of 486,790 SNP loci were obtained. Due to the small sample size included in this GWAS study, no SNP loci reached the significant difference level of genome-wide association studies (*P* < 5.0× 10^-8^), but 10 SNPs reached *P* < 5×10^-5^ (**Table 3**).

**Table 3:** Ten SNPs reached P< 5×10^-5^ in DN GWAS study.

SNP	Gene symbol	CHR	Minor allele	Major allele	DN(major/minor allele frequency)	DM(major/minor allele frequency)	P value	OR (95% CI)
rs6467788		7	T	C	0.40/0.60	0.72/0.28	6.67E-06	3.90(2.13-7.14)
rs825050	MYBPC1	12	T	G	0.90/0.10	0.61/0.39	5.85E-06	0.18(0.08-0.40)
rs149205645	AKAIN1	18	G	A	0.81/0.19	1.00/0	7.94E-06	
rs251418	PDE8B	5	T	C	0.97/0.03	0.75/0.25	1.30E-05	0.09(0.03-0.33)
rs200888	LOC100289473	20	T	G	0.50/0.50	0.79/0.21	2.39E-05	3.80(2.02-7.17)
rs12029233	LYPLAL1	1	A	G	0.69/0.31	0.93/0.07	2.57E-05	5.78(2.39-13.96)
rs4676864		3	A	C	0.91/0.09	0.65/0.35	2.64E-05	0.19(0.09-0.44)
rs2066405	USH2A	1	A	G	0.82/0.18	0.54/0.46	2.85E-05	0.25(0.13-0.49)
rs10859525	SOCS2	12	G	A	0.79/0.21	0.51/0.49	4.35E-05	0.27(0.14-0.52)
rs77481693		7	A	C	0.98/0.02	0.78/0.22	4.80E-05	0.08(0.02-0.36)

a CHR, chromosome; CI, confidence interval; OR, odds ratio; A, adenine; T, thymine; C, cytosine; G, guanine.

### Gut microbiota related variation in DN genetic susceptibility sites

The NHGRI GWAS Catalogue database was searched for genetic variants associated with the gut microbiota (16, 17), and the searched loci were identified in our DN cohort. No sites were found that reached the level of genome-wide significant association (*P* < 5.0×10^-8^). However, we still found 13 sites that were associated with DN, as shown in **Table 4**.

**Table 4:** The thirteen SNPs associated with DN with P values of<5×10^-2α^.

SNP	CHR	Position (hg19)	Risk allele	RAF in DN (%)	RAF in DM (%)	*P* value	OR(95%CI)	Gene Symbol
rs9600567	13	75871180	T	44.79	18.75	1.07×10^-4^	3.52(0.33-1.83)	LMO7
rs1422155	5	170892785	G	33.33	54.88	3.83×10^-3^	0.41(0.31-0.22)	RANBP17
rs17387919	7	24500681	C	6.25	18.75	8.83×10^-3^	0.29(0.50-0.11)	NPY
rs2140551	2	48595242	G	35.42	54.17	8.99×10^-3^	0.46(0.30-0.26)	STON1
rs7521798	1	206813581	C	15.62	5.21	1.82×10^-2^	3.37(0.54-1.17)	IL19
rs9972588	15	88868574	T	13.54	27.08	1.97×10^-2^	0.42(0.38-0.20)	ACAN
rs4277044	10	113075693	A	7.29	1.04	3.02×10^-2^	7.47(1.08-0.90)	TCF7L2
rs3746118	19	3762500	T	18.75	32.29	3.14×10^-2^	0.48(0.34-0.25)	APBA3
rs3747113	22	24321550	A	14.58	27.08	3.30×10^-2^	0.46(0.37-0.22)	SPECC1L
rs6551253	3	28371475	C	33.33	19.79	3.37×10^-2^	2.03(0.34-1.05)	ZCWPW2
rs2714053	11	123441355	A	32.29	46.88	3.88×10^-2^	0.54(0.29-0.30)	GRAMD1B
rs17836935	17	74179810	G	36.46	22.92	4×10^-2^	1.93(0.32-1.03)	RPL38
rs2294239	22	29053489	G	36.46	51.04	4.17×10^-2^	0.55(0.29-0.31)	ZNRF3

a CHR, chromosome; CI, confidence interval; OR, odds ratio; RAF, risk allele frequency; A, adenine; T, thymine; C, cytosine; G, guanine.

### Correlation between genotypes and clinical subphenotypes of DN

**Figure 1:**
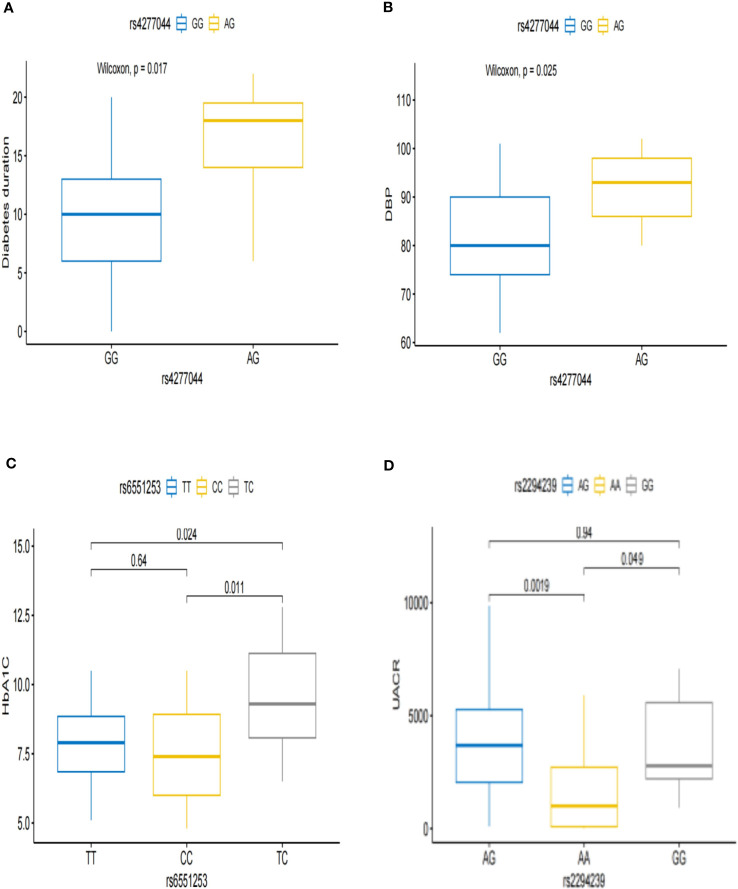
Associations between the genotypes and clinical subphenotypes of DN. **(A, B)** Patients with rs4277044-AG genotype had a longer diabetic duration and higher diastolic blood pressure than did patients with the rs4277044-GG genotype. **(C)** Patients with rs6551253-TC genotype had higher glycated hemoglobin than did Patients with the rs6551253-TT/CC genotypes. **(D)** The urine microalbumin-to-creatinine ratio was higher in patients with the rs2294239-GG/AG genotypes than in patients with the rs2294239-AA genotype.

Next, we analyzed the correlation between these 13 DN genetic variation sites related to intestinal microbes and clinical phenotypes and found that the risk genotype of TCF7L2 rs4277044-AG was associated with a longer diabetes duration and higher diastolic blood pressure (**Figures 1A, B**). The risk genotype of ZCWPW2 rs6551253-TC was associated with a higher level of glycosylated hemoglobin (**Figure 1C**). The risk genotypes of ZNRF3 rs2294239-GG/AG were associated with higher levels of urinary microalbumin-to-creatinine (**Figure 1D**).

### Microbial quantitative trait locus annotation

Data mining of the published microbial quantitative trait loci indicated that the risk genotypes of rs2294239 (ZNRF3) and rs3747113 (SPECC1L) in diabetic nephropathy were correlated with the abundance of Lactococcus, which is a microorganism that is beneficial to the human body (18, 19) (**Table 5**).

**Table 5:** Microbiome QTL annotations.

Genetic variant/risk allele in present study	Genetic variant/risk allele in GWAS Catalog	Variant annotation	Associated microbiome	*P value*	GWAS Catalog accession no.
rs7521798-C	rs7521798-C	intron_variant		4×10^-6^	GCST90006995
rs2140551-G	rs2140551-A	synonymous_variant		2×10^-9^	GCST90032517
rs6551253-C	rs6551253-C	intron_variant		3×10^-6^	GCST90007005
rs1422155-G	rs1422155-?	intron_variant		4×10^-6^	GCST90011577
rs17387919-C	rs17387919-?	regulatory_region_variant		2×10^-6^	GCST90011535
rs4277044-A	rs4277044-A	intron_variant		8×10^-6^	GCST90007003
rs2714053-A	rs2714053-G	intron_variant		5×10^-8^	GCST90027571
rs9600567-T	rs9600567-T	non_coding_transcript_exon_variant		5×10^-6^	GCST90006994
rs9972588-T	rs9972588-?	intron_variant		6×10^-6^	GCST008900
rs17836935-G	rs17836935-?	intergenic_variant		8×10^-6^	GCST90011571
rs3746118-T	rs3746118-?	upstream_gene_variant		7×10^-6^	GCST90011351
rs3747113-A	rs3747113-?	synonymous_variant	g_Lactococcus	3×10^-7^	GCST003221
rs2294239-G	rs2294239-G	intron_variant	g_Lactococcus	3×10^-6^	GCST003855

a g_, genus.

### Gut microbiota in patients with diabetic nephropathy and type 2 diabetes

**Figure 2:**
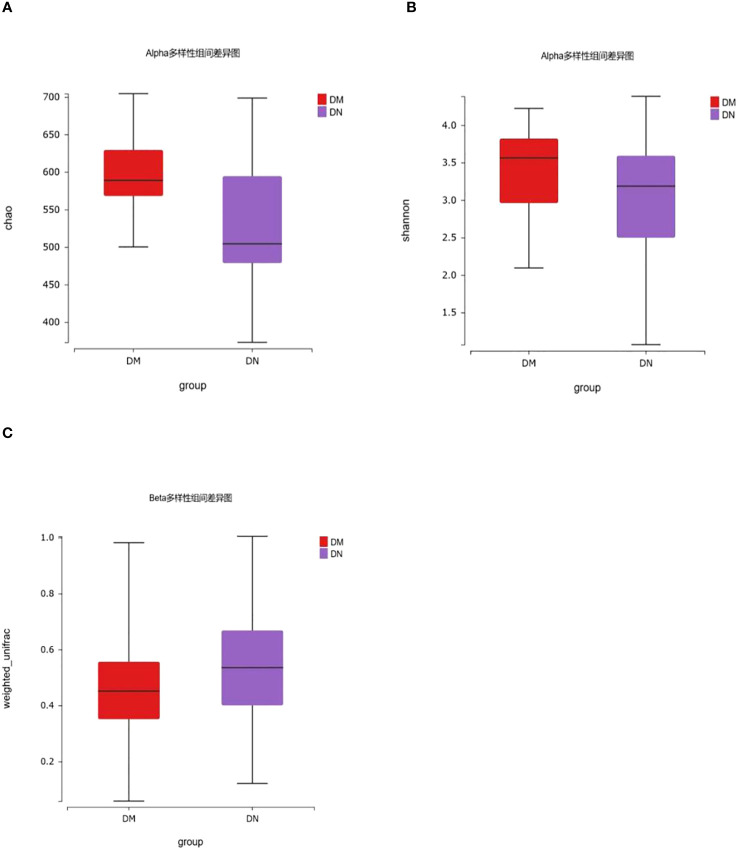
16S rDNA gene analysis in DM and DN groups. **(A, B)** The alpha diversity showed that gut microbiota richness of DN patients decreased (*P* = 5.38×10^-3^), while there was no significant difference in gut microbiota diversity between the two groups (*P* = 0.13). **(C)** β diversity showed that there were significant differences in microbial diversity between DN and DM groups (*P*= 1.60×10^-12^).

We included 50 patients with diabetic kidney disease confirmed by renal biopsy (DN group) and 50 patients with type 2 diabetes for more than 10 years without microvascular disease (DM group). The alpha diversity of bacterial communities was evaluated according to the Chao and Shannon indices. The Chao index was used to measure microbial species richness in a single sample, and the Shannon index was used to evaluate community diversity in a single sample. The results showed that compared with that in the DM group, the gut microbiota richness in the DN group was decreased (*P*=5.38×10^-3^), but there was no significant difference in gut microbiota diversity between the two groups (P=0.13). (**Figures 2A, B**). β diversity was used to compare the differences in species diversity among different samples, and the results showed that there were significant differences in microbial diversity between the DN and DM groups (P= 1.60×10^-12^) (**Figure 2C**).

**Figure 3:**
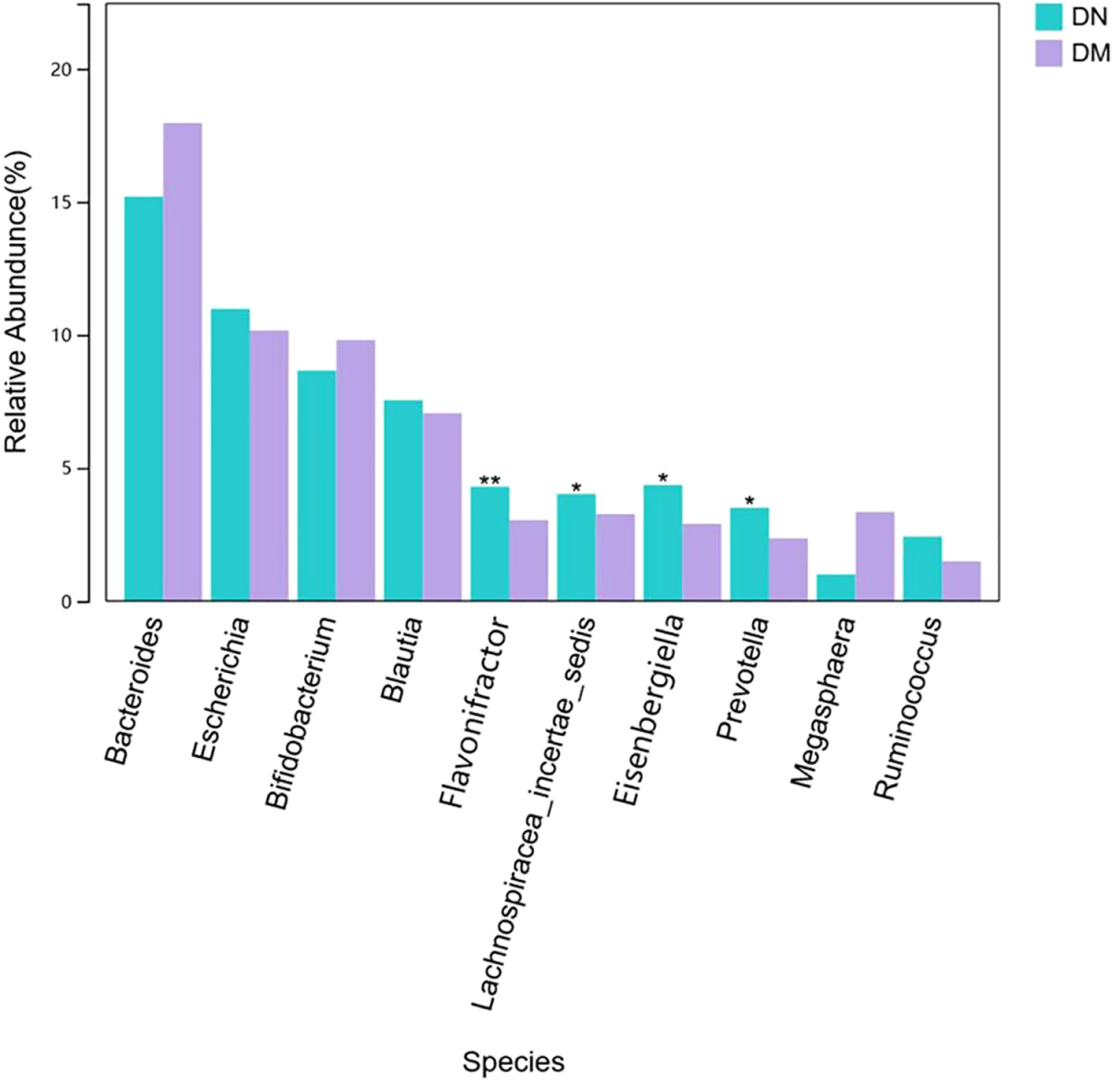
Bar chart of difference comparison of key species in gut microbiota (*P<0.05, **P<0.01).

To verify the clues suggested by the susceptible sites, we observed the differences in bacteria between the DN group and the DM group. We found that the relative abundance of Lactococcus in DN was 6×10^-6^ and that in DM was 1.7×10^-4^. There was a significant difference between the two groups (*P* = 0.04). However, the relative abundance of Lactococcus in the two groups was low, and the comparison of key species between the two groups showed that the abundances of Flavonifractor, Lachnospiracea_incertae_sedis, Eisenbergiella and Prevotella in the DN group were significantly increased compared with those in the DM group (**Figure 3**). However, no corresponding microbial quantitative trait loci were found in the genetic susceptibility locus of DN.

## Discussion

Host genome and gut microbiota composition have influenced the occurrence and development of many human diseases. Genetic variants associated with the microbiome are defined as microbial quantitative trait loci (QTLs). Recently, some studies have identified microbiome QTL in human diseases. For example, in inflammatory bowel disease, 5 functional genetic variants that were shown to be directly involved in gut bacterial processing (12). In IgA nephropathy, LYZL1 and SIPA1L3 risk genotypes are related to the decrease of Dialister and Bacilli and the risk genotypes of PLTP and AL365503.1 were associated with increased abundance of Erysipelotrichaceae and Lachnobacterium (13).

In this study, We searched for microbial QTLs in the GWAS Catalogue database and explored the relationship between the retrieved microbial QTLs and DN susceptibility and clinical subtypes in our DN genetic susceptibility locus screening cohort. In a cohort with 85 patients with DN and 107 patients with DM, we found that 13 loci were associated with DN susceptibility, and the genotypes of these loci were closely correlated with clinical subphenotypes. For example, the TCF7L2 risk genotype was associated with a long duration of diabetes and high diastolic blood pressure, the ZCWPW2 risk genotype was associated with a high level of HbA1c, and the ZNRF3 risk genotype was associated with an elevated urinary microalbumin-to-serum ratio. Studies of genotypic associations between risk alleles and clinical subphenotypes provide new insights into the etiology and mechanisms of diseases.

Through the study of genetic genes and the annotation of microbial QTLs, we found different microbiota in DN and DM and then identified the structure of the fecal microbial community by 16S rDNA sequencing. The risk genotypes of SPECC1L and ZNRF3 determined by genetic studies were correlated with Lactococcus. 16S rDNA sequencing confirmed that there were obvious differences in Lactococcus between DN and DM patients. Lactococcus is a probiotic, and probiotics play various roles in the human body, such as participating in the formation of the microbial barrier of the digestive tract, substance metabolism, nutrient transformation and biosynthesis, regulation of gastrointestinal immune function, and promotion of human growth and development (20). However, the abundance of Lactococcus in the fecal microbiota of the two groups was low, and the comparison map of the difference in key species between the two groups showed that the abundances of Flavonifractor, Lachnospiracea_incertae_sedis, Eisenbergiella and Prevotella in the DN group were significantly increased compared with those in the DM group. However, since no QTL of these microorganisms was found in our susceptibility genes, it was impossible to determine the influence of genes on these bacteria. In this study, we have tentatively demonstrated that host genetics have an impact on the gut microbiota, which plays an important role in both susceptibility and severity of disease.

Gut microbial composition is not only related to the environment, diet, disease, age, and sex but is also affected by host genetic factors, which will be beneficial to the accurate diagnosis and treatment of diseases (21). First, host genetics can affect the composition of the gut microbiota. Unlike variable factors such as diet, genetic factors are immutable factors. Therefore, we can determine whether the host is susceptible to the influence of DN risk microbiota through genetic analysis. Then, DN risk stratification can be conducted in the population, especially in the high-risk population, through genetic analysis and early intervention can be conducted in the high-risk population. Second, the relationship between genetic factors and the gut microbiota provides new insights into the pathogenesis of DN. In DN, the gut microbiota can interact with the kidney through the gut-renal axis (11), but the exact mechanisms and interactions with genetic factors are still unclear. Finally, this study provides new evidence for precision treatment and gut microbiota intervention in DN. Future studies integrating host genetics, gut microbiology and microbial metabolomics will be conducive to further elucidating the pathogenesis of DN and making accurate diagnoses and treatments.

There are some limitations to our study. First, the sample size included in our DN susceptibility gene study is small, so some microbiome-related variations may not be found in the DN genetic susceptibility sites. For example, Flavonifractor, Lachnospiracea_incertae_sedis, Eisenbergiella and Prevotella, which have significant differences and high abundance in the two groups, and their related genetic variations may be closely related to DN susceptibility. Second, at present, there is no extensive microbiome-related research on DN and even less research on DN-specific microbial QTLs. Therefore, many DN-related microbial QTLs have not been effectively annotated. Finally, fecal 16S rDNA sequencing has limited accuracy for microbial identification, and some microorganisms may not be effectively identified. In the future, we need to use more advanced technical means, expand the sample size of the study, and conduct multicenter and large-cohort joint studies to verify our results.

In conclusion, our microbial QTL genetic study showed that there were 13 loci closely related to the susceptibility and clinical subphenotypes of DN, and the risk genotype determined by genetic study was related to Lactococcus. Fecal microbial measurement also confirmed that this beneficial bacteria was significantly reduced in DN patients, which benefits the future accurate diagnosis and treatment of DN.

## Data availability statement

The data presented in the study are deposited in the Sequence Read Archive (SRA) repository, accession number PRJNA996574.

## Ethics statement

The studies involving humans were approved by The Biomedical Ethics Committee of Shanxi Provincial People’s Hospital. The studies were conducted in accordance with the local legislation and institutional requirements. The participants provided their written informed consent to participate in this study.

## Author contributions

XL: Conceptualization, Project administration, Writing – review & editing. JM: Data curation, Methodology, Writing – original draft. LG: Formal analysis, Writing – review & editing. WW: Methodology, Writing – original draft. RL: Project administration, Writing – review & editing.
